# Job Quality in the Late Career in Sweden, Japan, and the United States

**DOI:** 10.1093/geroni/igab046.1606

**Published:** 2021-12-17

**Authors:** Hugo Westerlund, Loretta Platts, Lawrence Sacco, Ayako Hiyoshi, Kevin Cahill, Stefanie König

**Affiliations:** 1 Stockholm University, Stockholm University, Stockholms Lan, Sweden; 2 Stress Research Institute, Department of Psychology, Stockholm University, Stockholm, Stockholms Lan, Sweden; 3 Stockholm University, Stockholm, Stockholms Lan, Sweden; 4 Örebro University, Örebro, Orebro Lan, Sweden; 5 Center on Aging & Work at Boston College, Chestnut Hill, Massachusetts, United States; 6 University of Gothenburg, Gothenburg, Gotlands Lan, Sweden

## Abstract

This paper examines job satisfaction and psychosocial and physical job quality over the late career in three contrasting national settings: Sweden, Japan and the United States. The data come from an ex-post harmonized dataset of individuals aged 50 to 75 years constructed from the biennial Swedish Longitudinal Occupational Survey of Health (SLOSH, 2006–2018, n=13936 to 15520), Japanese Study of Ageing and Retirement (JSTAR, 2006–2013, n=3704) and the United States Health and Retirement Study (HRS, 2006–2016, n=6239 and 8002). The job quality outcomes were physical labour, psychosocial working conditions (time pressure, discretion, pay satisfaction, job security) and job satisfaction. Random effects modelling was performed with age modelled with spline functions in which two knots were placed at ages indicating eligibility for pensions claiming or mandatory retirement. Interestingly, in each country, post-pensionable-age jobs were generally less stressful, freer, and more satisfying than jobs held by younger workers.

